# Scientific Items

**Published:** 1882-03-20

**Authors:** 


### SCIENTIFIC ITEMS.

**The Agricultural Ant of Texas.**—The existence of ants who carried on some of the processes of agriculture was known as far back as the time of Solomon, as proved by the well known passage in the Book of Proverbs, in which he praises their exceeding wisdom, and bids the sluggard to consider their ways; but as investigations of the habits of European ants led to the discovery that they none of them make provision for winter use, for the good reason that as they hibernate they do not require to do so, it came to be believed that Solomon had only adopted a popular fallacy; modern research has, however, proved that, after all, Solomon was correct, for there are two species inhabiting the countries bordering upon the Mediterranean that harvest seeds, and now we have from the other side of the Atlantic a full description of the mode of life and operations of farmer ants.

Mr. McCook, a Presbyterian minister of Philadelphia, provided by the liberality of his congregation with time and means for careful investigation, records that the *Pogonomyrmex barbatus* of Texas carries on what may be well and justly called "farming" like a true backwoodsman. As soon as a new colony is founded, the settlers proceed to clear the ground. This they do by biting through the stems of the grasses, allowing them to wither, and then pulling up the roots, and having cleared a circular space varying in diameter from one to ten feet, according to the strength of the community, this is afterwards kept free from all growth save of one particular species of grass, the seeds of which form their favorite food. It has been surmised that they sow the crop, but Mr. Cook's investigation failed to establish the proof of this; but they do not rely solely on the produce of their own clearing, but make diverging roads—in some cases upwards of 60 feet in length and 2 inches in width—into the surrounding jungle, and send out foraging parties in search of various grass seeds. These are borne home and deposited in their subterranean dwellings, the work being carried out from early morning till sunset, but with a rest of some hours during the heat of the day, when the busy workers retire under ground for protection from the sun's rays.—*Dru. Circular.*

**Flowers in Sleeping Rooms.**—The London Lancet makes the following remarks on the practice of keeping flowering plants in sleeping apartments:

The public are again warned against the use of flowers in sleeping apartments; and wonderful stories are told of the deleterious effects which have followed their presence in a limited atmosphere respired by invalids. Curiously enough, these appalling "instances" of the evil influences of plants do not for the most part apply to flowers. Nevertheless, we agree that it is safe to banish growing plants and flowers from bedrooms. They can do no good, and they may do some harm, if only by rendering the air of the apartment irritating to the delicate lining membrane of the breath organs. We are not disposed to endorse or accept the charge brought against plants and flowers generally, but it is well to err on the side of prudence; and although it cannot be denied that these embellishments form most pleasing objects for the eye, this advantage must be sacrificed, if, as alleged, they are injurious. There can be no doubt that some plants give off noxious emanations, and others may scatter particles which prove irritating; but are all vegetable growths thus injurious? However, as we have said, it is well to be over cautious. So flowers and plants must needs be banishsd, though we part with them with unfeigned reluctance.—*Drug. Circular.*

**Pulmonary Gymnastics.**—Dr. D. F. Powell, of LaCrosse, Wisconsin, writes to the New York Medical Record, as follows: "Ten years ago my chest measurement during full-forced inspiration was thirty-seven inches. By systematically inhaling and distendir g the air-cells as recommended by Niemeyer (and Smith, in the Record of Octocer 29, 1881), I have increased chest measurement to forty-four inches, and have wonderfully developed the pectoral and intercostal muscles. I write this to warn those who may adopt pulmonary gymnastics against inspiring air at too low a temperature, as I have on several occasions suffered from acute bronchitis, brought on by the forced inhalation of 'raw' cold air. I find that deep inspirations of pure air, at a temperature of from 60° to 80°, are of marked benefit in nearly all incipient lung diseases. I also freely use air medicated with carbolic acid, tar, iodine, bromine, nitrate of potash, etc., as indicated, and believe that no other treatment equals it as a remedial agent."—*Ibid.*

**Milk to Extinguish Petroleum.**—The first impulse of the inexperienced is to throw water upon burning kerosene oil, but the oil, being lighter than the water, rises upon it, and the only result is to make the fire spread. Several cases have recently been reported in the Metal Worker, where milk has proved an efficient extinguisher when every other means had failed. It may be doubted whether the well watered milk that reaches city tables would answer. The question also arises whether it would not be well to put a little milk in our lamps on filling them, so if they happen to explode, or are broken by a fall, the milk will be there, ready to act at once, without waiting for the milkman to come around.—*Boston Journal of Chemistry.*

**Effects of the Electric Light on the Eyesight.**—M. Nodier mentions in the Revue Scientifique, December 10, 1881, the instance of two naval officers, who, after some experiments with the electric light, were attacked with quite serious visual troubles; there were marked photophobia, slight conjunctivitis, lacrymation, contraction of the iris, and flying spots in the eyes.—*Drug. Circular.*

**A German** patent has been issued for a bottle made of twelve per cent. of silicum, which is said to resist the strongest acid. It is also recommended for the iron plates of zinc and iron galvanic batteries.

